# The Current Landscape of Antibody-based Therapies in Solid Malignancies

**DOI:** 10.7150/thno.52614

**Published:** 2021-01-01

**Authors:** Ashu Shah, Sanchita Rauth, Abhijit Aithal, Sukhwinder Kaur, Koelina Ganguly, Catherine Orzechowski, Grish C Varshney, Maneesh Jain, Surinder K Batra

**Affiliations:** 1 Department of Biochemistry and Molecular Biology, University of Nebraska Medical Center, NE, 68198, USA.; 2 Eppley Institute for Research in Cancer and Allied Diseases, University of Nebraska Medical Center, NE, 68198, USA.; 3 Fred and Pamela Buffett Cancer Center, University of Nebraska Medical Center, NE, 68198, USA.

**Keywords:** Antibodies, cancer, therapy, mechanisms of action, challenges

## Abstract

Over the past three decades, monoclonal antibodies (mAbs) have revolutionized the landscape of cancer therapy. Still, this benefit remains restricted to a small proportion of patients due to moderate response rates and resistance emergence. The field has started to embrace better mAb-based formats with advancements in molecular and protein engineering technologies. The development of a therapeutic mAb with long-lasting clinical impact demands a prodigious understanding of target antigen, effective mechanism of action, gene engineering technologies, complex interplay between tumor and host immune system, and biomarkers for prediction of clinical response. This review discusses the various approaches used by mAbs for tumor targeting and mechanisms of therapeutic resistance that is not only caused by the heterogeneity of tumor antigen, but also the resistance imposed by tumor microenvironment (TME), including inefficient delivery to the tumor, alteration of effector functions in the TME, and Fc-gamma receptor expression diversity and polymorphism. Further, this article provides a perspective on potential strategies to overcome these barriers and how diagnostic and prognostic biomarkers are being used in predicting response to mAb-based therapies. Overall, understanding these interdependent parameters can improve the current mAb-based formulations and develop novel mAb-based therapeutics for achieving durable clinical outcomes in a large subset of patients.

## 1. Introduction

A tremendous benefit to the cancer patients has been gained with advancements in therapeutic antibodies' development, initially introduced with the concept by Paul Ehrlich to target diseased cells using magic bullets 1. Primarily developed murine monoclonal antibodies (mAbs), while useful in mouse models, exhibit limited clinical utility due to their immunogenicity and inability to efficiently engage the human immune system's effector arm. However, advances in molecular and protein engineering technologies enabled therapeutic mAbs with lower immunogenicity, increased specificity and affinity, and efficient distribution through the vasculature to the tumor mass. After many years of research, better mAb-based formats targeting different approaches simultaneously, including bispecific antibodies (bsAbs), antibody-drug conjugates (ADC), and chimeric antigen receptor (CAR) T cells, have been developed 2. While some of these engineered mAb-based therapeutic agents received initial approval but were later discontinued due to severe toxicity, clinical data is awaited for many others.

Therapeutic mAbs exert direct anti-tumor effects via multiple mechanisms**.** A majority of therapeutic mAbs act by direct functional neutralization of target antigen on the tumor to inhibit tumor cell proliferation and metastasis, stromal cells to modulate tumor microenvironment, or immune cells for modulation of host immune response. Alternatively, therapeutic mAbs can opsonize tumor cells to facilitate their destruction via engagement of immune effector cells or components of the complement system, or a combination of both. In addition to these direct effects, mAbs have also been used as targeting vehicles for the delivery of cytotoxic agents like drugs, radionuclides, and toxins to malignant cells in an antigen-specific manner. However, the antibody-based drugs' efficacy in several malignancies is compromised due to the tumor heterogeneity and the complex landscape of molecular mechanisms driving immune exhaustion in the tumor microenvironment (TME). Considerable progress has been made in the development of histology and molecular imaging-based biomarkers to predict response to therapeutic mAbs. Nonetheless, the selection of patients who would derive benefit from mAb-based therapies remains a challenge.

This review provides an overview of various mechanisms underlying the anti-tumor activity of therapeutic mAbs and critical determinants of therapeutic resistance. We highlight the emerging approaches being employed to overcome these barriers by using combinational targeting, TME modulators, and Fc engineering. Moreover, we provide an update on currently present biomarkers for diagnosis and prognosis of anti-tumor therapeutic mAbs, critical in patient selection, and help develop mAbs with lasting therapeutic impact.

## 2. Mechanisms of Anti-tumor activity of therapeutic antibodies

**Figure 1:**
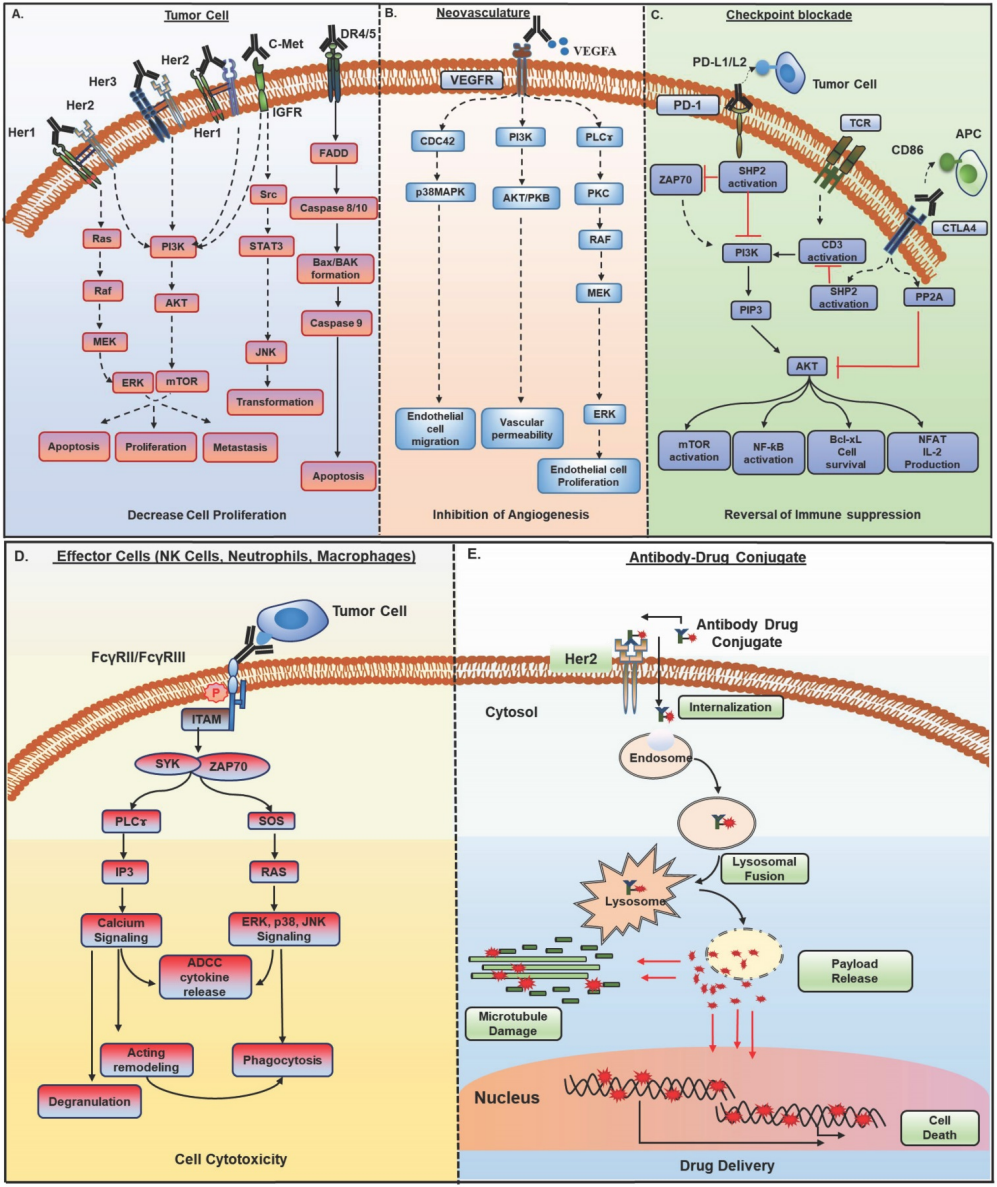
** Mechanisms of action of monoclonal antibodies (mAb) in cancer therapy**. Antibody-based therapeutic strategies (A-E) in solid tumors include both direct and indirect tumor cell killing. **A)** Antibodies acting by functional neutralization of receptor(s) on tumor cells. Antibody binding to overexpressed HER family receptors (HER1, HER2, HER3) on tumor cells, interfere with ligand binding or inhibit their homo- and hetero- dimerization with other HER family members and inhibit activation of downstream MAPK/ERK and PI3K/AKT signaling pathways that promote growth, migration, and proliferation of tumor cells. Antibodies against c- met receptor block STAT3/JNK and PI3K/AKT signaling and inhibit tumor cell transformation, survival, and proliferation. Recently developed class of agonistic antibodies to TNF superfamily death receptors DR4 and DR5 stimulate apoptosis through Bax/Bak and Caspase 9 pathway. **B)** Binding of antibodies to tumor vasculature receptors VEGFR1, VEGFR2, or their ligand VEGFA inhibits endothelial cell proliferation, migration, vascular permeability, and angiogenesis by interfering with PI3K/AKT, MAPK, and MEK/ERK signaling. **C)** Antibodies to immune checkpoint molecules, which include inhibitory (CTLA4) and co-inhibitory receptors (PD-1) present on immune cells or their ligands upregulated by tumor cells (PD-L1), reverse T cell exhaustion. Anti-PD-1 antibodies interfere with tyrosine phosphatase SHP2 recruitment and allow TCR (CD3) induced PI3K/AKT and MAPK signaling activation, cell survival, and proliferation. Anti-CTLA4 antibodies inhibit PP2A recruitment and CD3 dephosphorylation and activate PI3K/AKT, mTOR, NF-kB signaling pathways. **D)** Antibodies bound to tumor cells display antibody-dependent cell cytotoxic (ADCC) activity by engaging FcγR, present on the effector cells such as NK cells, neutrophils, and macrophages. This interaction induces ITAM phosphorylation and binding to tyrosine kinases ZAP-70 and SYK, which in turn activate PI3K and SOS. PIP3 generated through PI3K activation recruit BTK and PLCγ. RAS, BTK, and PLC activate downstream ERK, p38 and JNK signaling pathway along with calcium release from the endoplasmic reticulum (ER), which result in the release of cytokines and cytotoxic granules as IFNγ, perforin, and granzymes from NK cells and actin remodeling, which finally cause tumor cell apoptosis. Antibody bound tumor cells are recognized by neutrophils and macrophages and trigger oxidative burst and phagocytosis by neutrophils and macrophages, leading to lysosomal degradation of tumor cells. **E)** Antibody-drug conjugates (ADCs) possess specificity of mAb and cytotoxic potential of payload drug. ADCs bind to target antigen, get internalized, and undergo endocytic processing. Once in the cell, ADCs cleavage occurs, and the active cytotoxic drug is released into the cytoplasm where it inhibits microtubule polymerization and subsequently, tumor cell death. HER, human epidermal growth factor receptor; MAPK, mitogen- activated protein kinase; ERK, extracellular signal- regulated kinase; MEK, MAPK/Erk kinase 1/2; PI3K, phosphoinositide 3-kinase; SOS, son of sevenless homologue; STAT3, signal transducer and activator of transcription 3; JNK, c-JUN N- terminal kinase; DR4, death receptor 4; DR5, death receptor 5; Bax, Bcl-2-associated X protein; VEGFR1, vascular endothelial growth factor receptor-1; VEGFR2, vascular endothelial growth factor receptor-2; VEGFA, vascular endothelial growth factor A; FcγR; Fc gamma receptor; PIP3, phosphatidylinositol-3,4,5-trisphosphate; NK, natural killer; ITAM, immunoreceptor tyrosine- based activation motif; SYK, spleen tyrosine kinase; BTK, bruton's tyrosine kinase; PLCγ, phospholipase C gamma; RAS, rat sarcoma viral oncogene homolog; CTLA-4, cytotoxic T lymphocyte antigen 4; PD-1, program death-1; PD-L1, program death-1 ligand; SHP2, src homology phosphatase 2; TCR, t cell receptor; CD3, cluster of differentiation 3; mTOR, mammalian target of rapamycin; NF-kB, nuclear factor kappa-light-chain-enhancer of activated B cells.

Therapeutic mAbs induce anti-tumor effects via diverse mechanisms (**Figure 1**), which depend on the nature of the target antigen, target cell, and the nature of interactions between Fab and Fc regions of mAbs with target antigen and effector cells, respectively. The mechanism of action of therapeutic mAbs guides the clinical applications in terms of patient selection, disease setting, and combination therapies.

### 2.1. Functional neutralization of target antigen

Therapeutic mAbs directly targeting tumor cells or the non-tumor cells in the TME recognize a) growth factor receptors or their ligands; b) angiogenic receptors present on tumor vasculature or their ligands; c) immune checkpoint molecules. The anti-tumor activity of these mAbs depends on the expression levels and persistence of target antigen on tumor cells. These mAbs induce conformational changes, cause steric hindrance or facilitate internalization and downregulation of cell surface receptors, and abrogate downstream signaling **(Figure 1A and B)**. Most of the mAbs against growth factor receptors target a family of receptor tyrosine kinases (RTKs) as EGFR, HER2, HER3, and c-met, etc. These mAbs are approved for different cancers, including metastatic colorectal cancer (CRC), non-small-cell lung carcinoma (NSCLC), and squamous cell carcinoma (SCC). There are currently four mAbs approved against EGFR for clinical applications, with cetuximab being widely used alone or in combination with chemotherapy or radiotherapy 3. Similarly, HER2 and HER3 targeting mAbs are quite effective in HER2 positive breast and lung cancer patients 4. Although therapeutic mAbs against growth factor receptors EGFR and HER2 have shown promising results, their efficacy can be compromised due to the emergence of multiple resistance mechanisms attributable to the tumor's genetic and pathophysiological characteristics (detailed in section 3). Indeed, several reports have shown that overexpression of other growth factor receptors induces resistance to cetuximab therapy in metastatic CRC 5. Therefore, several clinical trials are evaluating the combination of mAbs targeting different receptors (described in section 4.1). Among mAbs targeting vasculature, bevacizumab (VEGF) was the first anti-angiogenic agent approved for clinical use in metastatic CRC patients, and after that, ramucirumab (VEGF-R1) received approval for various malignancies 4. Besides, mAbs against other growth factor receptors, including c-met, PDGFR, Ang-1, Ang-2, and IGF-1R, are being tested in clinical trials, and olaratumab (anti-PDGFRα) was approved in 2017 for soft tissue sarcoma patients.

In the last decade, therapeutic antibodies directly targeting immune checkpoint molecules [referred to as immune checkpoint blockade (ICB)] (**Figure 1C**) have captured the center-stage for cancer immunotherapy 6. Since the approval of anti- CTLA4 mAb, ipilimumab, for advanced unresectable melanoma in 2010, antibodies targeting immune checkpoint molecules have been approved for several malignancies 7. mAbs to another checkpoint molecule PD-1 or its ligand PD-L1 showed remarkable success in reversing the immune exhaustion. Two anti-PD-1 antibodies, pembrolizumab (keytruda) and nivolumab (opdivo), received FDA approval for various cancers, including metastatic melanoma, NSCLC, and RCC, in 2014. Other mAbs targeting different checkpoint molecules like LAG3 or CD223, BTLA (B and T lymphocyte attenuator), VISTA, and TIM3 are also being investigated to reduce immunosuppression, and few are in early phase clinical trials for various malignancies 8.

### 2.2. Engagement with effector cells

Therapeutic mAbs crosslink tumor cells and effector arm of the immune system via their antigen-binding domain (Fab) and Fc domain, respectively and elicit a range of effector functions. These include induction of antibody-dependent cell cytotoxicity (ADCC) through neutrophils and natural killer (NK) cells, antibody-dependent cell phagocytosis (ADCP) *via* macrophages, and complement-dependent cytotoxicity (CDC) by complement pathway of the immune system (**Figure 1D**).

#### Antibody-dependent cellular cytotoxicity (ADCC)

Therapeutic mAbs can induce ADCC activity by binding to various Fcᵧ receptors (FcᵧR) present on immune effector cells, including FcγRIIa (CD32) and FcαR1 (CD89) expressed exclusively on neutrophils and FcγRIIIa (CD16) on NK cells. Neutrophils exert cytotoxic activity by degranulation resulting in the release of proteases, cytokines, and tumor necrosis factor-alpha (TNFα) upon binding to opsonized target cells 9. The presence of high-affinity receptor FcαR1 (CD89) on neutrophils makes them potent effector cells for IgA antibodies 10. Therefore, isotype exchanged IgA version of IgG1 cetuximab exhibited potent *in vivo* ADCC activity against EGFR transfected Ba/F3 target cells 11. Similarly, drastic tumor reductions were observed with the engagement of neutrophils with tumor cells in the presence of anti-EGFR, HER2, CD30, and Ep-CAM antibodies of IgA isotype 9, 12 possibly through the release of chemoattractant Leukotriene B4 (LTB4), which causes increased neutrophil accumulation in the tumor microenvironment 13. Another study showed an increase in the neutrophil extracellular trap (NET) formation by engagement of FcαR with IgA antibodies 14. However, chronic granulomatous disease patients with no NET formation showed effective mAb-mediated tumor cell killing, suggesting the involvement of multiple mechanisms for mAb mediated tumor killing by neutrophils and that need to be further explored. Interestingly, a recent study has identified trogoptosis as a potential mechanism for neutrophil-mediated ADCC, which occurs by the formation of synapse between mAb-opsonized tumor cells and neutrophils via CD11b/CD18, resulting in the disruption of the target cell membrane 15. This killing is enhanced by targeting the CD47-SIRPα axis, which is an innate immune checkpoint pathway.

NK cells serve as critical components in tumor cells, killing through ADCC. Interaction of target bound mAb constant region (F_c_) with FcγRIIIA (CD16), and/or FcγRIIC (CD32c) present on NK cells induces ITAM (immune tyrosine-based activation motif) phosphorylation, ZAP-70 and Syk kinase-dependent activation of PI3K, NF-κB, and ERK pathways, which leads to both NK cell degranulation and signaling causing target cell killing (**Figure 1D**) 16. In addition, IFNγ secretion from NK cells facilitates the recruitment of cytotoxic T cells to lyse target cells 17, 18.

#### Antibody-dependent cellular phagocytosis (ADCP)

Antibody-mediated phagocytosis by macrophages has been best studied in hematological malignancies. Unlike NK cells, which express only FcγRIIIa, macrophages express all classes of Fc receptors (FcγRI, FcγRII, and FcγRIIIa). Macrophages are thus believed to be the major effectors of mAb-mediated therapy 19. The intricate relationship between macrophages and tumors complicates the understanding of macrophage's role as effectors for therapeutic mAbs. While macrophages have been traditionally viewed as vital cells for cancer immunotherapy, a high number of tumor-associated macrophages have been associated with adverse patient outcomes. The critical role of macrophages in mAb-mediated therapy became evident when their depletion resulted in decreased efficacy of anti-CD142 mAbs in preventing breast cancer growth and metastasis 20. Subsequently, different approaches have been used to augment ADCP activity by macrophages. For instant, mutations in the Fc region increased anti-Ep-CAM mAb binding to FcγRIIa and enhanced ADCP-mediated killing of LS180 adenocarcinoma cells. Similar results were observed with aglycosylated trastuzumab, which showed a 75% increased ADCP activity with low HER2 expressing cell lines 21. A recent study demonstrated that trastuzumab-mediated ADCP occurs *via* engagement of FcγRIIa and FcγRIII on human macrophages. Surprisingly, this study showed that macrophages displaying trastuzumab-mediated ADCP downregulated NK cell-mediated ADCC and anti-tumor immune response by CD8^+^ T cells due to PD-L1 and IDO upregulation by inflammasome activation 22. In fact, a combination of ICB with trastuzumab displayed a synergistic effect in the murine model, which provides a rationale for combining trastuzumab in combination with anti-PD-L1 mAb or IDO inhibitors in clinical studies. While macrophages have been traditionally viewed as vital cells for cancer immunotherapy, numerous studies have also demonstrated that a high number of tumor-associated macrophages (TAMs) correlate with adverse patient outcomes. Indeed, agonistic anti-CD40 mAb have shown effective clinical response in pancreatic cancer mainly through the reactivation of macrophage effector activity 23 and subsequently, several anti-CD40 mAbs have been developed and are in different clinical trials as monotherapy or combinations with chemotherapy, radiations or immunotherapy for various malignancies 24.

#### Complement-dependent cytotoxicity (CDC)

Therapeutic mAbs mediate complement-dependent cytotoxicity (CDC) *via* their interaction with soluble complement protein C1q through the Fc domain. This interaction activates the complement cascade culminating in the formation of a membrane attack complex (MAC) on the tumor cell surface leading to cell lysis. Although tumor killing by antibodies through CDC in a clinical setting is debatable, complement activation predominantly promotes the elimination of mAb-opsonized cells *via* ADCC and ADCP 25. Importantly, these diverse immune effector mechanisms are interlinked and can thus augment tumor cell killing in a synergistic manner 26.

Most therapeutic mAbs exert anti-tumor effects by multiple mechanisms of action that are interdependent and, therefore, might influence their anti-tumor activity**.** For example, extensive research on trastuzumab and cetuximab in non-clinical studies suggested mAb action beyond signaling inhibition, which include receptor downregulation, degradation (in case of cetuximab), decrease in angiogenic factors, reduced production of active truncated HER2 (p95) fragments via inhibition of HER2 cleavage (in case of trastuzumab) 27, 28, 29 and ADCC dependent killing of tumor cells 30. It can be argued that the induction of signaling and receptor downregulation on target cells might alter the ADCC activity of antibodies. Nonetheless, preclinical studies have shown enhancement of T cell response *via* effective DC presentation of mAb lysed target cells. Over the past decades, technological innovations in mAb engineering have allowed the generation of bispecific antibodies (bsAb) with novel functions where two binding specificities are linked in one mAb for encompassing their functional properties. Currently, 25 bsAbs targeting tumor heterogeneity, different growth factor receptors, multiple checkpoints, and angiogenesis are in the different phases of clinical development for solid tumors 31. Among these, bsAbs redirecting T cell effector function (biTE; bispecific T cell engager) through CD3 activation, independent of T cell receptor (TCR) engagement, towards tumor-associated target antigen(s), are the most emerging class in the field of immunotherapy and have been elegantly reviewed elsewhere 32. These modalities have displayed limited potency and greater toxicity in different preclinical and early clinical studies. Nevertheless, tremendous progress has been made on the optimization of existing formats for preferential tumor engagement and augmented effector function by cytotoxic T cells, with the ultimate goal of developing safer and effective drugs. Though bsAb catumaxomab targeting EpCAM and CD3 received approval based on the better response in a subset of cancer patients, it was recently discontinued due to toxicity. Nevertheless, clinical data obtained from bsAbs trials would guide further optimization for better drug safety and efficacy and might open new avenues for cancer treatment. Besides, bsAbs have been utilized for pretargeting which have been discussed in section 2.4 on radioimmunotherapy (RIT).

### 2.3. Antibody-drug conjugates (ADC)

The ability of mAbs for specific targeting and stability has fueled the development of antibody conjugates for the delivery of cytotoxic payloads to the tumor site and overcome off-site toxicity issues associated with chemotherapies 33, 34. ADCs exert their activity by specific binding to the tumor cell, internalization *via* receptor-mediated endocytosis, endosome formation, and fusion with lysosomes, thus creating an acidic and protease (cathepsin, plasmin, etc.) rich environment, resulting in ADC cleavage and cytotoxic payload release in the cytoplasm that in turn hinders replication machinery or microtubule assembly and subsequent cell death (**Figure 1E**). Several challenges exist with the successful development of ADC, including target antigen selection, mAb specificity and affinity, the payload potency and stability in circulation, and linker selection. More than 60 ADCs are in different clinical development phases with the latest approval of fam-trastuzumab deruxtecan-nxki for metastatic breast cancer patients 34.

### 2.4. Radioimmunotherapy

Radioimmunotherapy (RIT) exploits the ability of antibodies to target tumors in an antigen-specific manner for selective delivery of therapeutic radionuclide and localized release of cytotoxic ionizing radiations. RIT can target ionizing radiation to both overt and occult lesions and has been successful in treating hematological malignancies. However, this success has not been recapitulated in solid tumors due to several factors, including intrinsic radioresistance of tumor cells, hypoxic TME, and long circulating half-life of radiolabeled mAbs, which result in a dose- limiting myelotoxicity 35. Nevertheless, various approaches have been employed to improve the efficacy of RITs for solid tumors 36. These approaches include target antigen selection criteria, compatibility between mAb pharmacokinetics and decay characteristics of radionuclides, administration route, utilization of biological response modifiers to enhance delivery, and use of pretargeting which have been reviewed elegantly elsewhere 35, 37, 38. Accumulating evidence suggests that the therapeutic index of RIT can be synergistically enhanced by combination with radiosensitizers, chemotherapy, or surgery; clinical data along these lines is awaited from several trials 38-40. Further encouragement in the field comes from the recent FDA approval of 131I-8H9 radioimmunoconjugate (anti-B7-H3; burtomab/omburtamab) for pediatric neuroblastoma patients with CNS metastasis and many other ongoing late-phase trials with this radioimmunoconjugate. This strategy of intracompartmental delivery of RIT allows effective tumor targeting as compared to systemic injections. However, similar attempts with another such intracompartmental (intraperitoneal) delivery of 212Pb-TCMC (S-2-(4-isothiocyanatobenzyl)-1,4,7,10-tetraaza-1,4,7,10-tetra(2-carbamoylmethyl)cyclododecane)-trastuzumab in HER2 positive intraperitoneal cancers demonstrated moderate toxicity in phase 1 clinical study (NCT01384253), but no data has been published on clinical efficacy. Variations in clinical efficacy for intracompartmental RITs can be expected by considering other factors, including tumor histology and burden, diffusion, mAb internalization, tumor radiosensitivity, and the nature of the metastatic/primary lesion. One of the major limitations with RIT in many cancers has been the inability to deliver sufficient doses of radiations due to toxicity issues by prolonged radiation exposure to radiosensitive bone marrow. For this, *in vivo* pretargeted RIT (PRIT) approach has been used successfully to improve the pharmacokinetics of RITs 41. In this approach, the unlabeled mAb is administered first and allowed sufficient time to localize to the tumor site, followed by the administration of radioactive moiety-linked small-sized molecule, which then binds to tumor bound mAb. The small-sized radionuclides exhibit better tumor penetration and lower off-target radiation toxicity to radiosensitive normal tissues. PRIT approach has evolved over time from a biotin-streptavidin-based system to the use of bispecific antibodies (bsAb) recognizing both tumor antigen and hapten 42. In the latter approach, following the localization of cold bsAb to the tumor (and clearance from the circulation), radiolabeled-hapten is administered where it saturates the hapten binding site and thus allows highly selective targeting. Bivalent [(anti-CEA and anti- diethylenetriaminepentaaceticacid (DTPA)] and trivalent [two anti- CEA and anti-hapten histamine-succinyl-glycine (HSG)] bsAb (named as TF2) constructs have been evaluated in metastatic medullary thyroid carcinoma (MTC) and advanced lung cancer patients, respectively. In MTC patients, anti-CEA X anti-DTPA bsAb based PRIT showed a significant clinical response in 76% of the patients with progression-free survival (PFS) of 13.6 months and median overall survival (OS) of 43.9 months. While PRIT using TF2 and 177Lu-IMP288 peptide was tested in advanced CRC and lung cancer patients for dose optimization, the clinical data suggested safe administration in CRC patients, and lung cancer patients showed cohort wise variations in terms of TF2 tumor uptake and clearance. In addition, the reduction in pretargeting delay from 48 hours to 24 hours increased the tumor uptake of TF2. However, 7/9 patients died within one year of treatment and follow-up, which can be explained by differences in the histology of two tumor types. The success of the PRIT approach is dependent on several parameters, including the target, challenges in the development of humanized bsAbs, bsAb to hapten/peptide ratio, the specific activity of radionuclide, and the interval between administration of pretargeting mAb and radiolabeled hapten/peptide. Nonetheless, innovations in improving these parameters for better targeting and diminished toxicity could provide more comprehensive RIT options in solid tumors.

## 3. Obstacles associated with antibody-mediated therapies in cancer

A multitude of factors have contributed to the limited success of mAb-based therapies, including heterogeneous target expression due to genetic and epigenetic mechanisms, pathophysiological barriers in the TME that impact the delivery and distribution of antibodies, poor immune cell infiltration, ablation of mAb effector functions, and FcγR expression diversity and polymorphism 43, 44. Understanding these parameters is essential for the development of strategies to overcome resistance and develop improved mAb-based therapies.

### 3.1. Target antigen

The clinical response of therapeutic mAbs acting through functional neutralization of target antigens is mainly determined by target antigen location, persistence, and downstream signaling cascade. The primary challenge for mAb-based therapy is the heterogeneous antigen distribution in malignant cells and the differences in target gene copy number across patients; therefore, a single mAb suggested for targeting may not be effective in all patients 45, 46. A clinically relevant example for this is seen by a better response of cetuximab or panitumumab in metastatic colorectal cancer patients having higher EGFR gene copy numbers 45, 47. Secondly, tumor-targeting can be negatively impacted by alterations in target antigen, including downregulation and structural modifications, alternate transcription initiation, driver mutations acquired during prolonged chemotherapeutic or radiation treatment, which subsequently lead to epitope conformation switching, failure of recognition by antibodies, and therapy resistance 48, 49, 50. In addition, the therapeutic response of mAbs targeting growth factor receptors such as EGFR, HER2, and HER3, is also influenced by aberrations in genes associated with their respective signaling cascades. Numerous studies are now delineating tumor resistance mechanisms by investigating the genomic landscape of mAb treated tumors 51, 52. A recent study with cetuximab in colorectal cancer patients showed intrinsically resistant tumors harboring genetic alterations in downstream RAS/RAF signaling pathways. Resistance to cetuximab resulted from the selection and enrichment of genetically altered rare resistant sub-clones in the primary tumors, increased epithelial to mesenchymal transition (EMT), and decreased immune infiltration 53. In the context of anti-VEGF antibodies, therapeutic resistance is accompanied by the neovascularization triggered through pro-angiogenic factors secreted from the macrophages present in the TME 54. In addition, several studies have shown an association of anti-VEGF mAb therapy resistance with a significant increase in the number of macrophages or downregulation of VEGFR-1 and VEGFR-3 with concurrent upregulation of alternative angiogenic pathways 55, 56. Indeed, macrophage depletion during initial anti-VEGF therapy resistance prolonged survival in a mouse model of ovarian cancer 56. Improved understanding of these potential resistance mechanisms associated with function neutralizing mAb therapies might help design better clinical trials by facilitating improved patient stratification and combining agents targeting signaling pathways involved in resistance.

### 3.2. Antibody delivery

One of the major obstacles limiting the clinical application of therapeutic mAbs is their transport through the blood and insufficient delivery to the tumor. Understanding the fundamentals of transport and clearance mechanism is crucial to determine the amount of mAb permeating through the tumor and the time taken to reach the target site. Many mathematical models have been developed to understand mAb distribution through tumors by determining the effect of mAb diffusion, binding barriers, interstitial fluid pressure (IFP), convection, cellular trafficking, and pharmacology 57-59. These models recapitulated heterogeneous distributions observed *in vivo* and demonstrated that complex interplay between these parameters might explain insufficient and heterogeneous mAb delivery through the tumors.

**Figure 2:**
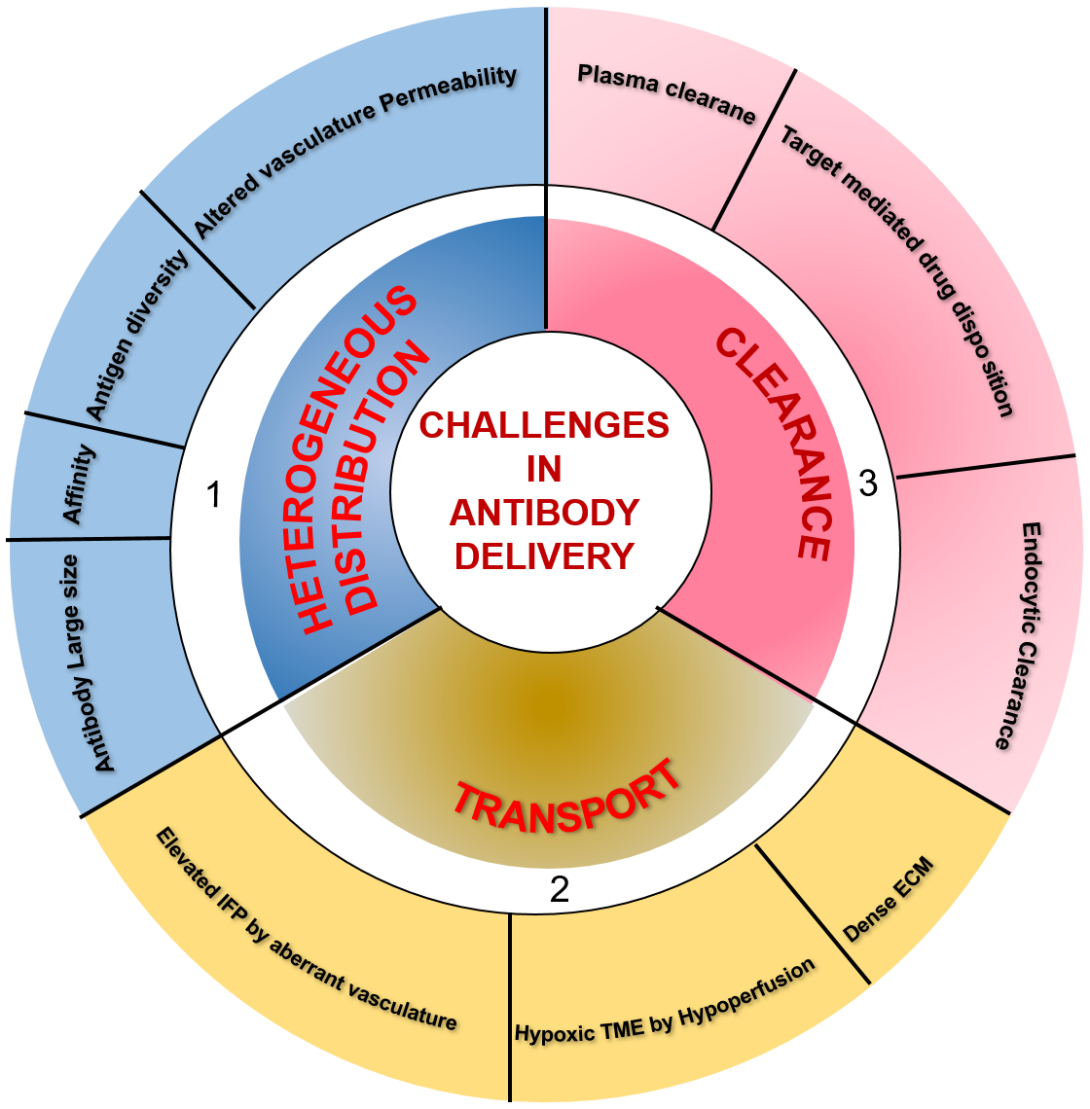
** Summary of factors influencing the delivery of therapeutic antibodies in solid tumors. 1)** A multitude of factors, including antibody large size and affinity, antigen diversity, and altered permeability of vasculature, leading to the heterogeneous distribution of systematically administered antibodies in tumor tissues. **2)** Hypoxia and pH dysregulation in the tumor microenvironment (TME) due to hypoperfusion, elevated interstitial fluid pressure (IFP) by aberrant vasculature, and dense extracellular matrix (ECM) influence the transport of therapeutic antibodies to intratumoral sites. **3)** The clearance of antibodies occurs from both inside and outside the tumor. Systemic clearance of antibodies from plasma hampers the concentration gradient required for antibody diffusion into the tumor. In lower antibody doses concerning a total amount of target antigen, antigen-antibody complexes undergo endocytic consumption and degradation (also called target mediated drug disposition effect). Local endocytic clearance through antibody internalization decreases antibody penetration to the tumor site.

TME is characterized by perturbed and inefficient vascular supply (hypoperfusion) and high interstitial fluid pressure (IFP) due to nonfunctional lymphatics that eliminate the pressure gradient required for transport of macromolecules via convection. Therefore, interstitial fluid flows outward at tumor margins, and the only diffusion drives mAb transport within the tumor. However, systemic clearance of mAb from the plasma disturbs the concentration gradient necessary for its diffusion into the tumor. Besides, mAb clearance through the endocytic pathway also influences mAb diffusion and distribution through the tumors. Therefore, the relative ratio between mAb transport by diffusion and loss by clearance determines the actual amount of antibodies reaching the tumor site. Additionally, tumor vasculature associated characteristics, including vessel permeability, rate of extravasation across capillaries, and intercapillary spacing, determine the amount of tissue perfusion. Leaky vessels in tumors and scare lymphatics result in elevated IFP, which accounts for limited extravasation and diffusion in tumor interstitium. Other factors influencing the mAb distribution and retention in the tumor are mAb size, affinity, and tumor physiology. The tumor cells and the associated soluble mediators influence both the immune and non-immune stromal components, including ECM, cancer-associated fibroblasts, and the deposited matrix proteins like fibronectin, collagen, and hyaluronic acid in the TME 60. The resulting dense stroma increases tissue rigidity and alters vascular permeability and diffusion through ECM, which hamper the transvascular and interstitial transport 61, 62 and penetration of macromolecular mAb drugs into the deep core of the tumor to recognize target-positive cells 63. Only 0.001-0.01% of administered mAb is accreted to the targeted site. Furthermore, hypoperfusion results in hypoxia and low pH, which cause immunosuppression by attenuating the cytotoxic potential of immune effector cells and impact the activity of mAbs inducing F_c_-mediated effector functions. Different parameters, including the large size of mAb, mAb affinity, tumor heterogeneity, kinetics of mAb transport and clearance, and nonfunctional lymphatics, influence targeting in solid tumors 59, 64 and are depicted in **Figure 2**.

### 3.3. Effector function impairment

**Figure 3:**
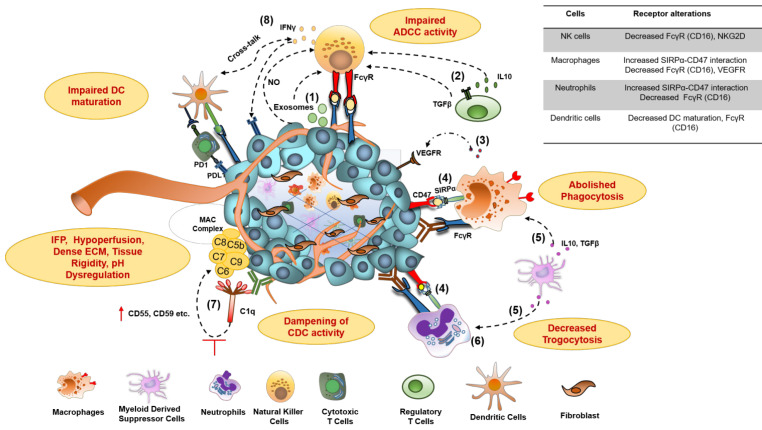
** Molecular mechanisms for impaired antibody therapeutic activity in cancer.** 1**)** Tumor-derived exosomes and Nitric Oxide (NO) secretion from tumor; **2)** membrane-bound TGFβ on Treg cells and secreted IL-10 contributes to impaired ADCC activity of NK cells, characterized by downregulation of NK cell activating receptor NKG2D and CD16 (FcγR) signaling and decreased IFNγ secretion; **3)** Secretion of proangiogenic cytokines from macrophages in TME, downregulate the expression of VEGFR1, VEGFR3, on vascular endothelium and resulting in resistance to anti-VEGF antibody therapies; **4)** Overexpression of don't eat me a signal (CD47) on tumors and its interaction with myeloid-specific checkpoint molecule SIRPα on tumor cells, contribute to dysfunction of phagocytic activity of macrophages and neutrophils through signaling via inhibitory ITIM motif; **5)** IL-10 and TGFβ produced from Myeloid-Derived Suppressor Cells (MDSCs) impair FcγR signaling and thus dampen the phagocytic activity of macrophages and neutrophils; **6)** Neutrophil-mediated antibody opsonized tumor cells killing, which involves CD11b/CD18 dependent conjugate formation and trogocytosis, abolishes by CD47-SIRPα axis;**7)** The overexpression of membrane-bound complement regulator proteins (mCRPs) such as CD55, CD59, etc. on tumor cells interfere with the binding of immune complexes (Antigen bound antibody) to C1q component of classical complement pathway and abolish Complement- dependent Cytotoxic (CDC) activity of antibodies; **8)** Dampening of antibody-induced tumor antigen-specific immune response results due to reduced IFNγ secretion from exhausted NK cells leading to insufficient DC maturation and decreased T cell activation. TGFβ, transforming growth factor β; IL-10, interleukin-10; IFNγ, interferon-gamma; SIRPα, signal regulatory protein α; ITIM, immunoreceptor tyrosine-based inhibitory motif.

Hypoxia and acidosis, the major hallmark of the TME, induce the secretion of TGFβ, IL-10, prostaglandin E2 (PGE2), kynurenine, adenosine (Ado), and several chemokines that alter the activities of various immune effector cells. These soluble mediators result in the attenuated cytotoxic potential of effector NK cells *via* NKG2D downregulation, exhaustion of effector T cells through the overexpression of inhibitory and co-inhibitory receptors, macrophage polarization towards suppressive M2 macrophages, and enrichment of immunosuppressive myeloid-derived suppressor cells (MDSCs) and T_regs_ in the TME (**Figure 3**). The co-culture of NK cells with MDSCs derived from melanoma, HNSCC, and breast cancer patients abrogated their FcᵧR-mediated signal transduction and ADCC activity through nitric oxide production 65. Another NK cell receptor, NKG2D, though not required for mediating ADCC, is critical for NK cell activation after binding to its ligands, major histocompatibility complex (MHC) class I-related chain A and B (MICA and MICB/MICAL-1 and MICAL-2), which are overexpressed on tumor cells. However, the ligands get proteolytically cleaved in the tumor cells to facilitate immune evasion from NK cell-mediated tumor cell killing 66.

Although well known for effective tumor-killing through ADCC and ADCP, myeloid effector cells frequently infiltrate to the tumors and display exhausted features, as evident from multiple studies 67. Crosslinking between tumor cell-associated CD47 and myeloid cell-specific checkpoint receptor signal regulatory protein-α (SIRPα) triggers negative signaling through immunotyrosine based inhibitory motif (ITIM), *via* recruitment of tyrosine phosphatases SHP-1 and SHP-2, and ultimately results in their phagocytic activity exhaustion (**Figure 3**) 15, 67, 68.

In the context of immune system suppression, mAbs to immune checkpoint molecules have established a significant and sustained improvement in tumor control, but a minority of patients' were responsive to these treatments, which dictate swinging efforts on identifying other targets to unleash or enhance the anti-tumor immune response. In this regard, targeting immunosuppressive TME has gained particular attention in recent years 69, 70. A recent study using intravital microscopy showed the removal of anti-PD-1 mAb from cytotoxic T cells and their transfer to surrounding PD-1^-^ TAMs, possibly through interactions of Fc domain with FcγRs on TAMs. Inhibition of FcγR by blocking mAbs resulted in complete tumor rejection in all animals by prolonging the interaction time between anti-PD-1 mAbs and PD-1 expressing CD8^+^ T cells 71. Recent studies have identified the presence of membrane-bound and soluble ectonucleotidases, including CD39 and CD73 on tumor cells, vasculature, and immune cells in regulating immunosuppressive adenosine generation in the TME that modulate immune response via suppression of effector cells, stabilization of immunosuppressive T_regs_, inhibition of macrophage maturation and DC activation 72, 73.

While the importance of a complement pathway in mAb-mediated tumor killing is well understood, contrasting studies from Wang et al. demonstrated, for the first time, the antagonistic role of complement for mAb therapy *in vivo* 74. Overexpressed complement pathway regulatory proteins (CRP), which can either be membrane-associated (mCRP) or secretory (sCRP), have been shown to dampen mAb-mediated CDC activity (**Figure 3**) 75, 76. For example, CD55, one of the overexpressed mCRPs on epithelial cells of advanced prostate cancer patients, regulates complement activation through on-off interaction with classical and alternate C3/C5 convertase and results in inadequate response to mAb-dependent killing through CDC. These findings were further validated by the experiments where blocking of CD55 and CD59 through mAbs enhanced the CDC activity of trastuzumab by 32-40% against breast cancer cells 77.

### 3.4. FcᵧR expressional diversity and polymorphism

FcᵧR mediated immune effector functions have gained particular attention in mAb-mediated cancer therapy, and continuous efforts are being made to augment mAb effector functions. MAbs binding to ITAM possessing FcγRs activates immune effector cells for cytotoxic activity, while their binding to ITIM containing FcγR dampens immune effector function. Being important regulators of mAb induced innate and adaptive immune response, FcγR receptors have recently been referred to as “antibody checkpoints” 78. The presence of different classes of human and mouse FcγR (FcγRI, FcγRII, and FcγRIII) and the existence of their allelic variants along with their disperse distribution on effector cells (**Table 1**) pose a challenge in evaluating the clinical activity of therapeutic mAbs.

The polymorphism of CD16, as well as inhibitory receptors, killer immunoglobulin-like receptors (KIR), on NK cells, has been directly associated with NK cells' response to mAb therapies. For example, two different FcγRIIIA receptor polymorphic variants 158F and 158V exhibit differences in binding affinity to human IgG1 with greater affinities observed for the FcγRIIIA 158V variant 79, 89. Furthermore, this effect has been well correlated with the differential response of NK cells in *in vitro* ADCC studies of head and neck squamous cell carcinoma (HNSCC) cell lines using NK cell populations from different individuals 90. Similarly, FcγRIIa 131H and 131R variants exhibit variation in binding affinity to mAbs and, in turn, altered ADCC activity. Cetuximab-treated colorectal cancer patients with the FcγRIIIA 158V/V and FcγRIIA 131H/H alleles had longer progression-free survival than 158F/F and 131R/R patients (5.5 months vs. 3.0 months, P = 0.005) 91. Breast cancer patients with FcγRIIA 131H/H and FcγRIIIA 158V/V had better clinical outcomes with trastuzumab 82. KIR, the highly polymorphic receptors, is present on NK cells and some T cell subsets, and their expression is highly diverse among the patient population. KIRs interaction with their ligands, class I HLA molecules, affects NK cell activation 92, which depends on the individual KIR genotype. Neuroblastoma patients with KIR/KIR ligand mismatch had better clinical outcomes with anti-GD2 mAbs, suggesting increased NK cell activation in the absence of KIR engagement 93. While the role of KIRs in mAb dependent NK cell activity is less understood, the KIR and FcγR genotype may stand out as the best prognostic marker for therapeutic mAbs possessing ADCC activity.

## 4. Strategies to overcome barriers

The knowledge accumulated regarding the potential resistance mechanisms against mAb-mediated therapies warrants conceiving innovative strategies for overcoming existing challenges and developing improvised anti-tumor mAbs that might improve clinical response in cancer patients. Understanding the complex interplay between mAb MOA, tumor cells, and the tumor immune milieu might pave the way towards developing combination therapies to unleash an optimal anti-tumor immune response.

### 4.1. Combinatorial targeting with therapeutic antibodies

**Figure 4:**
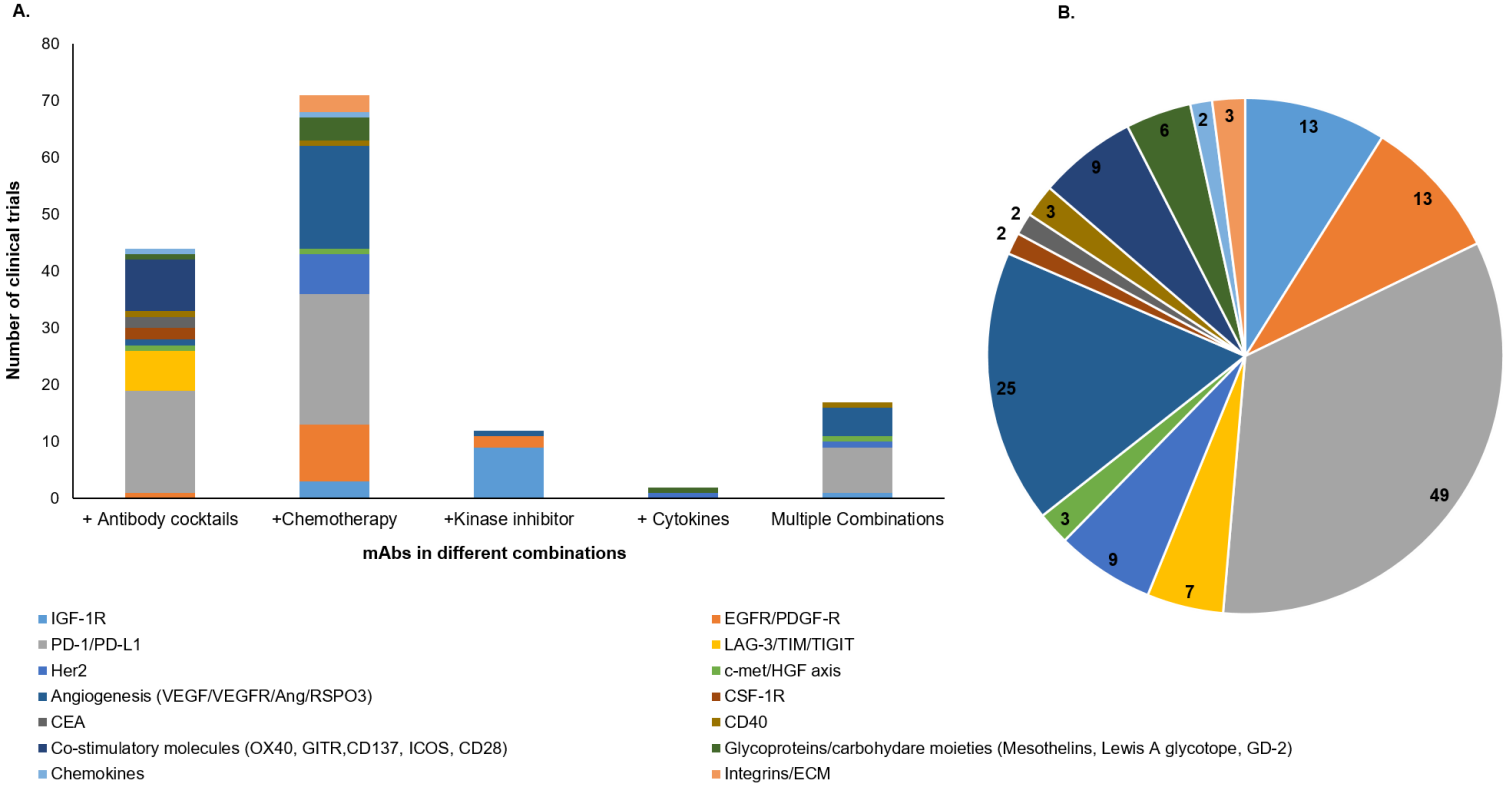
** Graphical representation of active clinical trials of mAb-based combinatorial targeting in solid tumors.** We searched for active clinical trials from the clinical trial site (https://clinicaltrials.gov/) with keywords solid tumors, antibodies plus chemotherapy, cytokines, and kinase inhibitors. These combinations are categorized as cocktails of mAbs, mAb+ chemotherapy, mAb+ kinase inhibitors, mAb+ cytokines, and multiple combinations where the mixture has more than two drugs. **A**. Each column of the graph represents the total number of clinical trials (y-axis) for each category of combination (x-axis). Different colors in each column represent target antigens for that category. **B.** Pie chart shows the number of mAb-based combination therapy trials for each class of target antigen(s) in solid tumors (colored separately).

To overcome the challenges associated with the negative impact of target antigen heterogeneity, several cocktails of therapeutic mAbs or their combination with different treatment categories, including chemotherapy, radiation, and kinase inhibitors; are being evaluated in the clinical studies 94. The combination of mAbs with chemotherapy or radiotherapy, have exhibited tremendous clinical success for various malignancies 95. Our search for ongoing clinical trials (www.clinicaltrials.gov) with different mAb-based combination therapies in solid tumors showed 150 trials, including mAb cocktails, mAb combination with chemotherapeutic drugs, cytokines, kinase inhibitors, or the multiple combinations where more than two categories of therapies are administered (**Figure 4**). Interestingly, the majority of clinical trials for these combination therapies are directed towards a combination of mAbs with chemotherapies and mAb cocktails (**Figure 4A**). Of note, most of the mAbs being evaluated in these combination therapy trials are targeted towards immune checkpoint molecules, angiogenesis, and growth factor receptors (**Figure 4B**). A recent review has classified and elaborated these therapeutic mAb cocktails as either homo- or hetero-combinations based on their target antigen 96. Homo-combinations are the mixture of mAbs targeting non-overlapping epitopes of the same antigen, while hetero-combinations target two or more discrete antigens that may be present on the same or different cell types. Homo-combination of anti-HER2 mAbs trastuzumab and pertuzumab was approved in combination with docetaxel for breast cancer patients 97. The clinical success of this combination therapy is attributed to their complementary mechanism of action and inhibition of potent ligand-dependent HER2-HER3 mediated signaling. Of note, all combination therapies did not show a synergistic effect, and few displayed severe toxicities leading to the inferior life quality of the patients. In the hetero-combination class, a cocktail of ICB nivolumab (anti-PD-1) and ipilimumab (anti-CTLA-4) got approval as a first-line treatment for untreated metastatic melanoma patients, and the latest clinical data showed up to four-year survival in 53% of patients receiving this combination therapy regimen 98. Later on, this cocktail was approved for low-risk renal cell carcinoma (RCC) and mismatch repair-deficient colorectal cancer (CRC) patients. The optimism for these mAb-based combination treatments in overcoming the therapeutic resistance across different malignancies is very high.

The quest for developing mAbs with multiple functions has led to the generation of bispecific antibodies (bsAbs), as discussed above. These modalities have opened new avenues for cancer therapy. Numerous bsAb constructs have been developed using knob-in-hole or limited Fab-exchange mechanisms and are in different preclinical and clinical stages of development 31. The direct comparison of bsAbs and mAb cocktails can be an arduous task as each treatment regimen has its advantages and limitations. bsAbs can be advantageous over mAb cocktails because of simultaneously targeting two different targets as a single molecule that will decrease the cost of manufacturing and clinical trials, provide higher binding specificity, and reduce off-target binding and toxicity. T cell-dependent bsAbs (anti-CD3 x anti-X; X- target antigen) allow the proximal presence of target cell and immune cell and, therefore, result in greater efficacy than expected from a combination of mAbs 32, 99. In addition, targeting two different receptors on the same tumor cell might overcome the development of resistance, usually associated with monospecific mAb therapies. However, bsAbs, due to their small size, exhibit a short half-life and may require repeated administration. The pharmacodynamics (PD) of each part of bsAb construct differs in association with the biology of each target, which might pose a challenge in drug development. Also, bsAb constructs restrict the targeting to a single combination of two antigens, unlike mAb cocktails, where random combinations can be tailored for both dose and administration sequence in various malignancies. Therefore, to date, a plethora of clinical trials are based on mAb cocktails in solid malignancies. However, the drug development process for combination therapies is cumbersome. Regulatory approvals need rationalization based on preclinical and clinical studies, which can substantiate the better therapeutic index for cocktails over monospecific therapies while bsAb require single regulatory approval. There are several clinical trials testing bsAbs in combination with ICB mAbs, which might raise questions regarding the therapeutic index of bsAbs. Nevertheless, continuous innovation in the field results in a series of bsAb constructs with a better half-life and improved therapeutic index. Data from various bsAb trials will guide in developing better formats and efficient clinical trial designs.

### 4.2. Enhancing mAb delivery

Various components of the TME, including dense stroma, abnormal tumor vasculature, and increased interstitial fluid pressure (IFP), impede the movement of therapeutic mAbs to the tumor site. It is hypothesized that reprogramming TME would improve mAb delivery and function effectively. Dense ECM in the TME is majorly constituted of hyaluronic acid and collagen and, therefore, approaches over the last two decades have focused on ECM targeting by the administration of pegylated hyaluronidase, reduction in collagen crosslinking by Lysyl oxidase (LOX) targeting or collagen synthesis through TGFβ inhibition, and decreasing fibrosis by vitamin D receptor ligand in different tumor xenografts 100. However, only some of these studies have evaluated the effect of these agents on mAb delivery. For example, Eikenes et al. showed a two-fold increase in the mAbTP3 uptake via a 45% reduction in IFP upon collagenase treatment in osteosarcoma xenograft studies 101. Similarly, the administration of collagenase in human ovarian cancer xenografts lowered tumor IFP and displayed a two-fold increase in trastuzumab binding on the tumor surface. While collagenase treatment enhanced mAb penetration by 4 hours from the predicted time, hyaluronidase treatment did not improve mAb uptake 102. In contrast, prior studies with human osteosarcoma xenografts have shown the effect of hyaluronidase in increasing mAb penetration by reducing tumor IFP. The difference in observations from these two studies can be linked to the heterogeneous composition of the fibrotic matrix in the two tumor models and variations in the route of hyaluronidase delivery. Intratumoral administration of hyaluronidase may have allowed for the better penetration of mAb. To date, most advanced ECM targeting is based on pegylated human hyaluronidase (PEGPH20) and is currently being evaluated in a clinical trial in combination with anti-PD-L1 mAb for gastric cancer patients. In contrast, a combination of PEGPH20 and pembrolizumab was withdrawn, though the reasons are not clearly mentioned. In addition, with the purpose of improving mAb delivery in solid tumors, attention is being given to cancer stroma targeting (CAST) using non-internalizing ADCs targeting endothelium 103. For example, one of the ADC has been developed by conjugating mAb targeting insoluble fibrin (IF), generated in the stroma through malignancy induced blood coagulation, to drug MMAE. The drug released can overcome hindrance by stroma due to small size and target cancer cells 104.

A growing body of evidence suggests that re-engineering TME through vascular normalization by the use of anti-angiogenic agents improves the efficacy of cancer immunotherapy. However, these effects were found to be dose-dependent since low doses of anti-angiogenic agents showed limited effects. In contrast, high doses resulted in excessive vessel pruning with unfavorable tumor perfusion and drug delivery. A comprehensive review on the normalization of tumor vasculature and ECM modulation can be referred to, for an in-depth understanding of re-engineering tumor TME as a promising therapeutic opportunity 105. Overall, these studies emphasize the significance of tumor ECM and IFP modulation for enhancing therapeutic mAbs delivery to the tumor site.

### 4.3. Effector cell modulation

Reprogramming of both immune effector functions and TME milieu is essential for achieving durable clinical responses with mAb-based therapies in solid malignancies. ADCC by NK cells contribute significantly to the therapeutic effects of anti-tumor mAbs, and impaired NK cell function in the TME can limit their therapeutic efficacy. Efforts directed to identify and target the factors responsible for the dysregulated activity of NK cells have yielded promising results. For example, remarkable clinical and immunological responses were achieved in HNSCC and CRC patients treated with the combination of cetuximab and immunomodulatory reagent lenalidomide, stimulating NK cell proliferation and activation 106. Likewise, gastric cancer patients treated with the combination of cetuximab or trastuzumab and adoptive NK cell therapy showed significant benefits 107. However, extensive studies with larger size patient cohort are warranted to develop these combination therapies further. Besides, there is an upsurge in the development of 2^nd^ and 3^rd^ generation of existing mAbs (biobetters) possessing a high affinity for FcγRIIIA and improved ADCC activity through genetic engineering and Fc afucosylation 108, 109. Efforts have been made to augment the ADCC activity of NK cells by combination with MMP inhibitors, cytokines, or radiation 110-112.

Improving myeloid cell effector functions is another avenue for enhancing the efficacy of therapeutic mAbs. Several antagonistic mAbs have been developed against CD47 or SIRPα to reverse the suppression of macrophages and neutrophils and augment their effector function. Anti-SIRPα mAb alone was ineffective in inhibiting tumor growth in a human SIRPA knock-in mouse (SRG) model. It provided significant tumor reduction in combination with tumor opsonizing rituximab or vorsetuzumab (anti-CD70) mAbs, with no effect on tumor-infiltrating myeloid cells composition 113. Several clinical studies are assessing mAbs against the CD47-SIRPα axis, particularly in combination with existing therapeutic mAbs 113, 114.

Anti-angiogenic mAb combination with ICB stimulated tumor immune response by normalizing the tumor vasculature. Different strategies have been used for re-educating the tumor microenvironment through stromal depletion for enhanced mAb uptake 63. Recently, mAbs generated against both membrane-bound and soluble forms of CD39 and CD73 promoted DC maturation and macrophage activation by blocking adenosine generation and led to an augmented anti-tumor response in various malignancies. Interestingly, concomitant blockade of CD39 and CD73 in T cells from healthy donors and breast cancer patients displayed a potent reversal of adenosine-mediated T cell inhibition and augmented anti-tumor response 115. Based on these observations, several clinical trials have been designed to evaluate the combination of anti-CD39/CD-73 mAbs with FDA approved ICB molecules. **Table 2** enumerates the strategies used to improvise the existing therapeutic mAbs for modulating the TME and their respective applications across various malignancies.

### 4.4. Fc Engineering

MAb X-ray crystal structures have increased the understanding of how Fab and Fc regions work in conjunction with binding and effector functions. The variation in the length of the hinge region separating Fab and Fc and the number of interchain disulfide bonds forms the basis for the differences in the conformation of Fab and Fc in human IgG subclasses and their binding to different Fcγ receptors. In addition, the mAb Fc domain glycosylation at N297 in motif Asn297-Ser-Thr of the CH2 domain is critical for mAb interaction with various receptors and mediating effector functions 120, 121. Therefore, the approaches to augment mAb effector functions include changing the isotype to increase engagement with immune components and altering IgG glycosylation patterns for enhancing interaction with FcᵧR on effector cells.

#### Altering FcᵧR engagement

It is conceivable that the engineering of the Fc domain for maximizing interaction with activating receptors with a concomitant reduction for FcγRIIB binding can enhance the therapeutic efficacy of most mAbs. This can be supported by the fact that trastuzumab and rituximab exhibited reduced efficacy in FcγR deficient mice and enhanced ADCC and better tumor killing in FcγRIIb deficient mice 122. In contrast, therapeutic mAbs antagonistically targeting TNFR superfamily members, death receptor DR4, DR5, or agonistically targeting CD40, engage inhibitory FcγRIIb receptor 123, 124 to exert their anti-tumor activities. However, the stimulatory activity of these mAbs depends on the distance of the epitope from the membrane, which determines the Fc accessibility and levels of FcγRIIb expressing cells in the TME 125, 126.

In the context of mAbs targeting immune checkpoint molecules, FcγR engagement, and ADCC activity have gained popularity in recent years 127, 128. Impressive data obtained with anti-CTLA4 mAbs of IgG1 and IgG2 isotype in the FcγR humanized mouse model highlight the importance of activating FcγR in immune response regulation *via* checkpoint molecules. These mAbs, upon binding to CTLA4 present on tumor-infiltrating T_reg_ cells, induced ADCC activity, thereby resulting in tumor-infiltrating T_reg_ cells depletion and enhanced tumor regression. In contrast, engineered mAbs lacking ADCC potential had reduced anti-tumor activity 85. It is important to note that this work, along with many previous studies, assumes the presence of effector cells expressing higher levels of activating FcγR in infiltrating immune cell population to induce ADCC and T_reg_ depletion 85, 129, 130. However, B16 melanoma failed to respond to anti-CTLA4 therapy due to a lack of CTLA4 expression on target cells and a reduced number of FcγR expressing effector cells. Of note, the higher response rate of anti-CTLA4 mAbs in patients with CD16V158F polymorphism suggested the critical role of FcγR engagement in mediating therapy response. Likewise, anti-PD-L1 mAb isotypes engaging activating FcγRs enhanced their therapeutic efficacy by altering the myeloid cells in TME while anti-PD-1 mAbs activity was negatively regulated by both activating and inhibitory FcγR 131. This study also highlighted the importance of epitope in determining FcγR mediated response by anti-PD-1 mAbs. On the contrary, a very recent study demonstrated that the anti-tumor activity of anti-PD-L1 mAbs in MC38 and CT26 syngeneic colon adenocarcinoma models was independent of FcγR engagement. However, a reduction in tumor-infiltrating myeloid cell populations was observed upon mAb treatment 131, 132. Overall, these studies warrant critical epitope selection in developing mAbs to this class of molecules for achieving profound clinical responses.

#### Fc glycoengineering

The type of glycan moieties attached to the Fc portion results in different Fc conformations, and possibly one out of these many conformers result in the Fc domain binding to FcγR 133. Other glycan structures are observed in the Fc portion of therapeutic mAbs, including G0F, G1F, G2F, hM3F, M6, etc. The pattern of mAb glycosylation is dependent on many factors, such as the host cell used for mAb production, culture conditions, glucose, dissolved oxygen, pH, and temperature 134. Therefore, an inconsistent glycan pattern poses a major challenge in therapeutic mAb manufacturing. Several attempts have been made to glycoengineer mAbs for a homogeneous glycan profile and incorporate different modifications, including aglycosylation, afucosylation, and galactosylation, for altering their immune effector function and increasing their therapeutic efficacy 135, 136. Recent studies have also suggested the role of hypergalactosylation in inducing changes to the CH_2_ domain and increased FcγRIIIa binding 137, but its effect on biological activity remains unexplored. Multiple approaches for the production of glycoengineered mAbs include the development of mAb-producing cell lines that lack or have reduced expression of α1, 6-fucosyltransferases, and β1, 4-N-acetylglucosaminyltransferase III (GnTIII) for the addition of bisecting GlcNAc to recombinant mAbs for reduced core fucose content. Recently, Thomann et al. demonstrated that *in vitro* Fc galactosylation performed in four different mAbs enhanced their ADCC activity significantly, while galactosylation on afucosylated mAbs did not change their ADCC activity 138. These results indicated the importance of afucosylation as a major determinant of ADCC. These studies also showed that increased FcγR binding might not alter mAb effector function, suggesting the possibility of other molecules or mechanisms bridging mAb binding to FcγR and its effector function. Overall, glycosylation plays a critical role in the effector functions and, thus, serves as a critical and modifiable determinant in fine-tuning the efficacy of therapeutic mAbs.

## 5. Biomarkers of antibody-mediated therapies

The limited success of therapeutic antibodies in many clinical trials has been partially attributed to the sub-optimal selection criteria of patients in terms of disease aggressiveness, poor prognosis, and target antigen expression. Therefore, identifying the predictive biomarkers could help select patients likely to benefit most from these targeted therapies and evaluate novel combinations with chemotherapy, immunotherapy, or anti-angiogenic agents. Traditionally, estrogen and progesterone have been used as biomarkers for trastuzumab therapies in breast cancer patients. FDA has approved cetuximab and panitumumab for CRC patients harboring wild-type RAS gene 139. Similarly, concerted efforts are being made to identify predictive biomarkers for different therapeutic mAbs with a particular focus on ICB therapies in various malignancies, including breast cancer, NSCLC, ovarian cancer, and SCLC for the optimal patient selection, which will eventually translate into improved clinical outcomes 139-141. The emerging technologies being used in biomarker studies include immunohistochemistry (IHC), multiplex fluorescence, and extensive genomic profiling for tumor mutation burden (TMB) and microsatellite instability (MSI). However, several studies have pointed out the limitations of existing assays for assessing biomarker(s) expression levels probably due to differences in methodologies across studies, inter-patient tumor variations though having similar histology, and different histological features of tumors 142. Consequently, the concept of companion diagnostics has emerged in the clinical setting, where the same diagnostic mAb is utilized as a therapeutic agent. The companion diagnostics help stratify the patients based on target expression and accurately predict the response to particular targeted therapies. Several companion diagnostic kits for mAbs, including trastuzumab (HER2), cetuximab (EGFR), pembrolizumab (PD-L1), and atezolizumab (PD-L1), are in clinical use.

A greater understanding of tumor genetics and inter- and intra-tumor heterogeneity warrants the identification of novel molecular markers and develop novel mAb-based therapies based on target expression profiles. For example, though farletuzumab targeting folate receptor α isoform (FRα) showed clinical efficacy in phase II trial of FR-positive ovarian cancer patients, phase III clinical data in advanced-stage platinum-resistant patients did not meet the prespecified end-point criteria for progression-free survival (PFS) 143. In addition, it may also be attributed to inappropriate patient selection as FR expression levels were not considered at the time of recruitment. Histology based biomarker expression studies have failed to address these tumor heterogeneities due to the dependence on tumor section being used for sampling. Therefore, efforts to develop novel imaging-based biomarkers continue, which are noninvasive and take in to account the whole tumor mass and *in vivo* receptor expression 144. The current tumor imaging methods include fluorescence imaging, computed tomography (CT), ultrasound imaging, magnetic resonance imaging (MRI), single-photon emission computed tomography (SPECT), and positron emission tomography (PET). Various mAbs, including cetuximab, bevacizumab, panitumumab, and trastuzumab have been conjugated with near-infrared fluorescent probe IRDye800CW and evaluated for imaging in preclinical studies 145, 146 based on which some mAbs are in different phases of clinical trials for imaging in glioma and pancreatic cancer. An FR-imaging agent 99mTc-etarfolatide (EC20) was used in the selection of patients for FR-targeted therapies. In fact, a lot of attention is being given to positron emission tomography (PET) based imaging due to high sensitivity and quantitative imaging properties, and many imaging probes for PET are under development. Accumulating evidence show the association of HER3 receptor upregulation with the emergence of anti-HER2 mAb therapeutic resistance in advanced breast cancer patients. Therefore, efforts to identify therapy-resistant patients by developing a PET imaging biomarker to monitor HER3 receptor expression changes have been made, and 89Zr-radiolabeled mAb (GSK2849330) is in a clinical trial for advanced solid tumor patients 147. However, the optimal use of imaging agents mandates antibodies with better penetration and short half-life. Therefore, currently, low molecular weight affibody-based PET imaging agents to monitor HER3 expression has been evaluated in breast and gastric cancer xenografts. Besides, bevacizumab conjugated quantum dots (QDs), the potential nanoparticles, were developed for both *in vitro* and *in vivo* imaging in human breast cancer xenografts in mice for determining mAb delivery and accumulation in the tumor 148. The QDs-bevacizumab would certainly help to address the challenges of aberrant tumor vasculature and the delivery of antibodies. Overall, imaging-based biomarkers would allow for improved identification of residual disease and help develop novel antibodies or mAb-based combination therapies for their targeting and patient stratification, thus improving clinical responses.

## Conclusions and future perspectives

The remarkable progress achieved with mAb-based anti-cancer therapies in the last ten years has been made possible by continuous innovations in molecular engineering, extensive knowledge accumulated on target biology, understanding the mechanism of therapeutic mAbs, and a greater appreciation of the immunosuppressive pathways operating in the tumor milieu. A combination of unique approaches being used by mAbs for tumor killing, including engagement of immune effector arm, modulating host immune response, and cytotoxic payload or radionuclide delivery, has proven useful in providing durable responses in cancer patients. However, knowing which mechanism will be effective in the clinical scenarios for a particular malignancy remains a challenge. In addition, target antigen persistence and induction of novel genomic alterations in a subset of tumor cells pose a real challenge with the success of mAbs in cancer therapy 53, 149. For this, several clinical trials are evaluating the combination therapies based on mAb cocktails or their combination with chemotherapy, radiotherapy, or cytokines (**Figure 4**). In fact, bsAb development is growing at a fast pace for dual targeting in different cancers. However, attempts are warranted for increasing their therapeutic index 31. Studies should be conducted to understand the intrinsic and extrinsic resistance mechanisms of mAb-based therapies. Contribution of altered target expression to therapeutic mAb resistance was evident in a recent study that demonstrated the acquisition of ADCC resistance in breast cancer cell lines to cetuximab and trastuzumab *via* reduced expression of EGFR, alterations in cell adhesion molecules involved in tumor-immune cell synapse, via epigenetic modifications 150. Epigenetic changes by HDAC inhibitors have recently been shown to modulate the response to immunotherapy in triple-negative breast cancer patients 151. However, extensive research is required to delineate the contribution of such epigenetic changes in target downregulation and simultaneous impact on mAb-mediated effector functions.

Beyond impeding the mAb delivery to the tumor mass, pro-tumorigenic TME impacts the quality and quantity of effector components, limiting effective FcγR engagement 59, 114, 128, 152. Strategies to enhance mAb penetration into the tumor by using anti-stromal agents (CAST therapy) or TME modulation through vasculature normalization and revert effector cell exhaustion for unleashing the potential of therapeutic mAbs in mediating anti-tumor immune responses are underway 66, 100, 104, 112, 113. While ICB agents have shown promising responses in the patients with various malignancies, the patient population size displaying durable responses remains small. Many clinical trials are evaluating their combination with either anti-angiogenic agents or TME modulators to achieve improved anti-tumor responses in a greater proportion of the patients 24, 115, 153. Another bottleneck for the anti-tumor mAbs that target the immune effector arm is finding the right balance in activating and inhibitory FcγR. It is important to note that significant efforts to engineer the Fc domain for fine-tuning this balance are underway, as exemplified by the efforts to engineer agonist mAbs to death receptors and immunostimulatory CD40 molecule (107). Extensive efforts have focused on glycolengineering and isotype switching to modulate FcγRs mediated effector functions 121, 138. Nevertheless, obtaining better effector response *in vitro* may not always forecast the clinical performance because the simultaneous presence of all the FcγRs in patients might influence the outcome of mAb-mediated therapies. To address these challenges, a recent study by Hussain et al. used whole blood samples for evaluating mAbs in functional assays. However, no significant association was observed between the FcγR genotype and mAb response 154. Nonetheless, HH/VV donors showed a better response in terms of IFNγ release, corroborating the importance of FcγR polymorphism in predicting clinical response to anti-tumor mAbs 82, 89, 155. Further work should focus on combining FcγR genotyping with *in vitro* assays for predicting clinical response with mAbs. Considerable progress has been made towards personalized medicine by developing diagnostic and predictive biomarkers based on genomic technologies, multiplex IHC, and molecular imaging tools. However, identification and validation of novel biomarkers for better patient stratification are warranted. The integration of diagnostic and prognostic biomarkers with clinical trial designs may help in the development of personalized mAb-based combination therapies and may improve clinical management and outcomes.

Overall, unraveling the intricate mechanisms that limit the efficacy of mAb-mediated therapies in cancer will help design strategies and identify novel pathways that can be targeted to overcome resistance and improve the effectiveness of mAb therapeutics in clinics. Combating effector arm exhaustion, TME modulation, and Fc engineering for improving effector functions are promising steps towards enhancing the therapeutic potential of mAbs. There is high optimism that durable clinical responses in a broad set of patients are achievable by rationalizing these combinations.

## Figures and Tables

**Table 1:** Distribution of Human and Mouse Fcγ receptors, their variants on effector cell subsets and antibody binding selectivity

FcγR	Motif	Variants^a^	Distribution^b^	Subclass selectivity^c^	Effector function^d^
**Human FcγR**					
FcγRI (CD64)	ITAM	NA	Monocyte, Macrophages, Neutrophils (inducible), Dendritic cells (inducible)	IgG1, IgG3, IgG4, mIgG2a,**mIgG2b	ADCP
FcγRIIA (CD32A)	-	H131, R131	Monocytes, Macrophages, Neutrophils, Dendritic cells	IgG1, IgG2, IgG3, IgG4	ADCP
FcγRIIB (CD32B)	ITIM	I232, T232	B cells, Dendritic cells, Monocytes, Macrophages	IgG1, IgG3, IgG4	Immune inhibition
FcγRIIC (CD32C)	-	Q57, *	NK cells (inducible), Neutrophils, Monocytes	IgG1, IgG2, IgG3, IgG4	ADCC
FcγRIIIA (CD16)	ITAM	V158, F158	NK cells, Macrophages, Neutrophils, NK cells, Dendritic cells	IgG1, IgG2, IgG3, IgG4	ADCC
FcγRIIIB (CD16B)	GPI anchor	**NA1(R36N65D82V106) NA2(S36S65N82I106), SH	Neutrophils	IgG1, IgG3	Immune inhibition
FcRn		NA	Endothelial cells, epithelial cells	IgG1, IgG2, IgG3, IgG4	Antibody recycling & transport
**Mouse FcγR**					
FcγRI (CD64)	ITAM	NA	DC, Monocytes, Macrophages	mIgG2a, mIgG2b	ADCP
FcγRIIB (CD32B)	ITIM	NA	B cells, DC, Neutrophils, Monocytes, Macrophages	mIgG1,mIgG2a, mIgG2b, IgG1	Immune inhibition
FcγRIII	ITAM	NA	NK, DC, Monocytes, Macrophages, Neutrophils	mIgG1,mIgG2a, mIgG2b	ADCC
FcγRIV	ITAM	NA	DC, Monocytes, Macrophages, Neutrophils	mIgG2a, mIgG2b, IgG1	ADCP
FcRn	NA	NA	Endothelial cells, epithelial cells	mIgG1,mIgG2a, mIgG2b, IgG3	Antibody recycling & transport

Notes: a. Data for the existence of FcγR variants is adapted from 79-82. b. FcγR distribution on different effector cells is compiled from 83-86. c. Antibody selectivity data is compiled from 85, 87, 88. d. Effector function data is compiled from 84-86. * Stop codon, *** NA1 and 2-neutrophil specific antigen 1 and 2, **m= denotes for mouse antibodies. NA, not available; ITAM, immunotyrosine based activation motif; ITIM, immunotyrosine based inhibitory motif; DC, dendritic cells; NK, natural Killer; ADCC, antibody dependent cell cytotoxicity; ADCP, antibody Dependent cell phagocytosis

**Table 2:** Overview of strategies for enhancing the efficacy of antibody-based therapies

	Agents	Strategy	Cancers	Reference
1.	Anti-MICAL-1 antibodies	Reactivating NK cell function	Melanoma	66
2.	Cytokines IL-2 and IL-15	Reactivating NK cell function	Head and Neck cancer	112, 116
3.	Anti-KIR Antibody (Lirilumab) in combination with ICB	Reactivating NK cell function	Solid tumors (advanced and refractory)	NCT01714739, NCT01750580
4.	IL-15 in Combination with ICB	Activation of immune system	Solid tumors (refractory)	NCT03388632
5.	Anti-CD40 agonist antibodies (ChiLob7/4) Anti-CD40 plus Tremelimumab	Direct cytotoxic effects on tumor cells Reprogramming of APCs	Solid tumors Melanoma	24 NCT01103635
6.	Anti-CSF-1 antibody (PD0360324/Lacnotuzumab) Anti-CSF-1R antibody (Emactuzumab/Cabiralizumab/ SNDX-6352)	Sensitization to ICB *via* decreased infiltration of macrophages and MDSCs	PC, BC, Melanoma, Ovarian, malignant neoplasms, RCC, NSCLC, Billiary tract cancer	70
7.	Antibodies against CD47-SIRPα axis	Acquired vasculogenic ability by the macrophages in the TME Enhanced trogoptosis by neutrophils	Solid tumors	15, 114, 117
8.	CXCR2 inhibition in combination with ICB antibodies	Inhibit trafficking of MDSCs to tumor site	Pediatric sarcomas	69
9.	IL-2 in combination with anti-CTLA4 antibody	Expansion and differentiation of effector T cells	Melanoma	NCT01480323
10.	Anti-CD39/CD-73 antibodies in combination with ICB	Reversal of adenosine mediated T cell exhaustion	Solid tumors	115
11.	Anti-VEGF/VEGFR2 antibody in combination with ICB	Vascular normalization, high endothelial venules (HEVs) formation, immune stimulation and decreased recruitment of immunosuppressive T_regs_	PC, BC, CRC	118, 119

Notes: This table summarizes the list of strategies being used to normalize tumor vasculature, decrease tumor-suppressive myeloid cells, and enhancing the cytotoxic activity of antibodies. PC, Pancreatic cancer; BC, Breast cancer; CRC, Colorectal cancer; RCC, Renal Cell Carcinoma; NSCLC, Non-small-cell lung carcinoma; IL-2, Interleukin-2; IL-15, Interleukin-15; NK, Natural Killer; DC, Dendritic cells; ADCC, Antibody-dependent cell cytotoxicity; ICB, Immune checkpoint blockade; APC, Antigen-presenting cell; MDSCs, Myeloid-derived suppressor cells; TME, Tumor microenvironment; CSF-1R, colony-stimulating factor receptor-1, CSF-1, colony stimulating factor-1; KIR, killer immunoglobulin receptor.

## Abbreviations

ADC: antibody-drug conjugates
ADCC: antibody-dependent cell cytotoxicity
ADCP: antibody-dependent cell phagocytosis
Ado: adenosine
CAR-T: chimeric antigen receptor t cells
CDC: complement-dependent cytotoxicity
CRP: complement pathway regulatory proteins
ECM: extracellular matrix
EMT: epithelial to mesenchymal transition
GnTIII: β1,4-N-acetylglucosaminyltransferase III
ICB: immune checkpoint blockade
ITAM: immunoreceptor tyrosine-based activation motif
ITIM: immunoreceptor tyrosine-based inhibitory motif
KIR: killer-cell-immunoglobulin-like receptor
MDSC: myeloid-derived suppressor cells
NK cell: natural killer cell
PGE2: prostaglandin E2
TME: tumor microenvironment
VEGF: vascular endothelial growth factor
